# Asymmetry of Anticipatory Postural Adjustment During Gait Initiation

**DOI:** 10.2478/hukin-2014-0056

**Published:** 2014-10-10

**Authors:** Koichi Hiraoka, Ryota Hatanaka, Yasutaka Nikaido, Yasutomo Jono, Yoshifumi Nomura, Keisuke Tani, Yuta Chujo

**Affiliations:** 1 School of Health and Human Services, Osaka Prefecture University, Habikino city, Osaka, Japan.; 2 Graduate School of Compremensive Rehabilitation, Osaka Prefecture University, Habikino city, Osaka, Japan.

**Keywords:** gait initiation, anticipatory postural adjustment, asymmetry, center of pressure

## Abstract

The purpose of this study was to investigate the asymmetry of anticipatory postural adjustment (APA) during gait initiation and to determine whether the process of choosing the initial swing leg affects APA during gait initiation. The participants initiated gait with the leg indicated by a start tone or initiated gait with the leg spontaneously chosen. The dependent variables of APA were not significantly different among the condition of initiating gait with the preferred leg indicated by the start tone, the condition of initiating gait with the non-preferred leg indicated by the start tone, and the condition of initiating gait with the leg spontaneously chosen. These findings fail to support the view that the process of choosing the initial swing leg affects APA during gait initiation. The lateral displacement of the center of pressure in the period in which shifting the center of pressure to the initial swing phase before initiating gait with the left leg indicated by the external cue was significantly larger than that when initiating gait with the right leg indicated by the external cue, and significantly larger than that when initiating gait with the leg spontaneously chosen. Weight shift to the initial swing side during APA during gait initiation was found to be asymmetrical when choosing the leg in response to an external cue.

## Introduction

Gait initiation is the transition between an upright stance and a steady-state gait (Caderby et al., 2013; Mickelborough et al., 2004). Anticipatory postural adjustment (APA), contributing to the forward progression of the body (Brenière and Cuong, 1987), occurs before the onset of the initial swing of the leg during gait initiation. The center of mass moves in an anterior direction and towards the initial stance leg during gait initiation, but the displacement does not occur until approximately 300 ms after activation of the tibialis anterior muscle (Elble et al., 1994). On the other hand, displacement of the center of pressure (COP) begins with an increase in the tibialis anterior muscles, which is considered to be the onset of gait initiation (Mann et al., 1979; Elble et al., 1994), indicating that the COP must be the appropriate parameter for observing APA during gait initiation.

The COP moves to the initial swing side and backward (S1 period) initially, moves to the initial stance side subsequently (S2 period), and finally moves forward (S3 period) during gait initiation (Hass et al., 2004). The S1 period has been highlighted in previous studies, because the S1 period is abnormal specifically in patients with Parkinson’s disease who frequently have difficulty in gait initiation (Burleigh-Jacobs et al., 1997; Halliday et al., 1998; Dibble et al., 2004; Okada et al., 2011-a). The posterior displacement of the COP during the S1 period is derived from the decreased activity of the triceps surae muscles and increased activity of the tibialis anterior muscle (Elble et al., 1994). The lateral displacement of the COP to the initial swing leg in the S1 period must be due either to the activity of the triceps surae muscle in the initial swing leg (Elble et al., 1994; Mickelborough et al., 2004) and/or to changes in the activity of the hip abductor and adductor (Kirker et al., 2000).

One of the recent issues in APA during gait initiation is how humans choose the initial swing leg. Preference of the initial swing leg has been found to affect APA before rapid single stepping and during gait initiation; the duration of APA, the displacement, and the velocity of the center of gravity during APA before a rapid single step with the preferred leg have been found to be larger than those with the non-preferred leg (Yiou and Do, 2010), and ground reaction force under the initial swing leg during APA during gait initiation with the non-preferred leg is larger than that with the preferred leg (Dessery et al., 2011). However, to the best of our knowledge, there have been no reports on the difference in APA during gait initiation when initiating gait with the left leg and that when initiating gait with the right leg, and thus, we address this issue in the present study.

The initial swing side of gait initiation is consistent from trial to trial in able-bodied humans and in PD patients without freezing of gait (FOG), but is variable between trials in PD patients with FOG (Okada et al., 2011-a; Okada et al., 2011-b), indicating that FOG is partially related to the process of choosing the initial swing leg for gait initiation. Furthermore, errors in APA when the leg for initiating gait is chosen by a start cue are more frequent than when initiating gait with the preferred leg (Cohen et al., 2011), indicating that the process of choosing the initial swing leg affects APA during gait initiation. When humans initiate gait with the leg spontaneously chosen, the process of choosing the initial swing side is minor. In contrast, when the initial swing leg is determined by an external cue immediately during gait initiation, an explicit choosing process is needed, and the gait initiation strategy changes when the initial swing side is predetermined (Henriksson and Hirschfeld, 2005). Thus, in the present study, we compared APA when initiating gait with the preferred leg indicated by a start tone (P condition), APA when initiating gait with the non-preferred leg indicated by a start tone (NP condition), and APA when initiating gait with the leg spontaneously chosen (NI condition), to test the hypothesis that APA during gait initiation is affected by the process of choosing the initial swing leg for gait initiation.

## Material and Methods

### Participants

The participants were 11 healthy males aged 31.0 ± 2.0 years. According to the Waterloo Footedness Questionnaire-Revised (WFQ-R), 9 of these participants were right-footers (Elias et al., 1998). This experiment was approved by the ethics committee of the Osaka Prefecture University.

### Measures

The participants were instructed to stand still with their feet closed on a force transducer (1G06/I-B; Nihon-Denki-Sanei, Tokyo, Japan) measuring the COP. A 180-cm long walkway with a width of 60 cm was placed in front of the transducer. Signals from the transducer were digitized at a sampling rate of 1 kHz using an A/D converter (PowerLab 800/S ADInstruments) and stored in a PC. The test-retest reliability of COP displacement and COP velocity during postural sway in standing was confirmed in a previous study (Lin et al., 2008). The intraclass correlation coefficients for these parameters were equal to or more than 0.80.

### Procedures

The participants were asked to load their weight on both feet equally during maintaining stand still on the transducer. The participants initiated gait in response to a start tone and continued to walk at their preferred speed to the end of the walkway. An experimenter monitored the COP online and delivered the start tone when the COP displacement in stance was small and stable. A high-frequency (1kHz) start tone instructed the participant to initiate gait with the right leg (R condition), a low-frequency (100 Hz) start tone instructed the participant to initiate gait with the left leg (L condition), and a middle-frequency (200 Hz) start tone instructed the participant to spontaneously choose the leg for gait initiation (NI condition). Ten trials were conducted for each start tone condition. The start tone conditions were randomly ordered trial by trial.

### Data analysis

**Figure 1:**
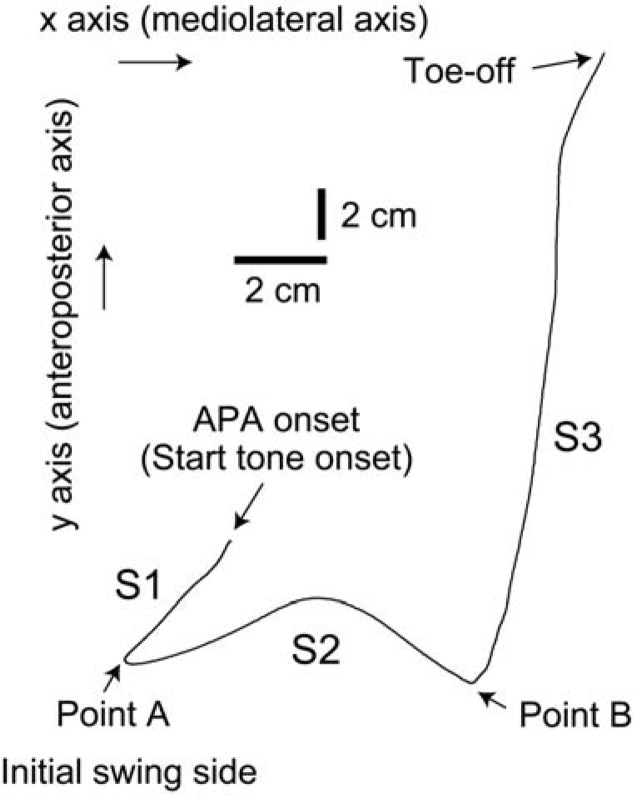
A bird’s-eye view of the COP trajectory during APA during gait initiation. The participant initiated the gait with the left leg moving ahead to the top of the figure. Points A and B are landmarks distinguishing the S1, S2, and S3 periods

The APA was divided into 3 periods (Hass et al., 2004): the S1 period, beginning with the start tone and ending with the COP located in the most posterior and lateral position toward the initial swing side (Point A); the S2 period, beginning with Point A and ending at the point at which the direction of the COP changed from the lateral direction to the anterior direction (Point B); and the S3 period, beginning with Point B and ending with the toe-off of the initial swing leg (Figure 1). The onset latency of the S1 period, and the duration, x displacement, y displacement, x velocity, and y velocity of each period were estimated (Hass et al., 2004). One analysis tested the difference in dependent variables among the L, R, and NI conditions (asymmetry analysis), while another tested the difference in dependent variables among the P, NP, and NI conditions (leg preference analysis). The preferred leg was defined as the leg most frequently swung first in the NI condition. One-way repeated measures analysis of variance (ANOVA) was conducted on the asymmetry analysis and leg preference analysis data sets. When ANOVA revealed a statistical difference, a post-hoc test, Bonferroni’s test, was carried out. The alpha was 0.05.

## Results

### Preferred initial swing leg

The preferred initial swing side in the NI condition was the right side in 7 participants. The initial swing side was consistent across the 10 trials in the NI condition in 10 participants.

### Onset and duration of APA

**Figure 2:**
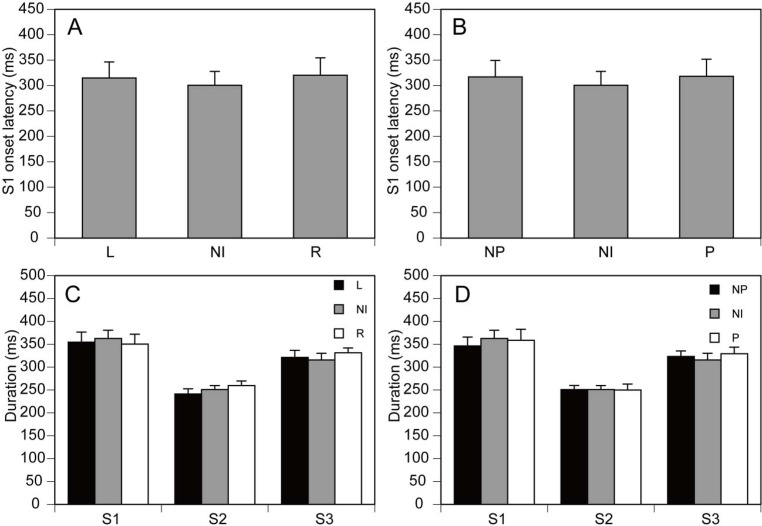
The onset latency of the S1 period (A) and the duration of each period (C) for the asymmetry analysis and for the leg preference analysis (B, D). Bars indicate means, and error bars indicate standard errors of the mean

The onset latency of the S1 period was not significantly different among the L, R, and NI conditions [F(2,20) = 0.663, p = 0.526] (Figure 2A) nor among the P, NP, and NI conditions [F(2,20) = 0.613, p = 0.552] (Figure 2B). There was no significant difference in the duration of the S1 [F(2,20) = 0.440, p = 0.650], S2 [F(2,20) = 0.957, p = 0.401], or S3 period [F(2,20) = 1.491, p = 0.249] among the L, R, and NI conditions (Figure 2C). There was also no significant difference in the duration of the S1 [F(2,20) = 0.831, p = 0.450], S2 [F(2,20) = 0.004, p = 0.996], or S3 period [F(2,20) = 1.064, p = 0.364] among the P, NP, and NI conditions (Figure 2D).

### Asymmetry analysis of the COP

**Figure 3:**
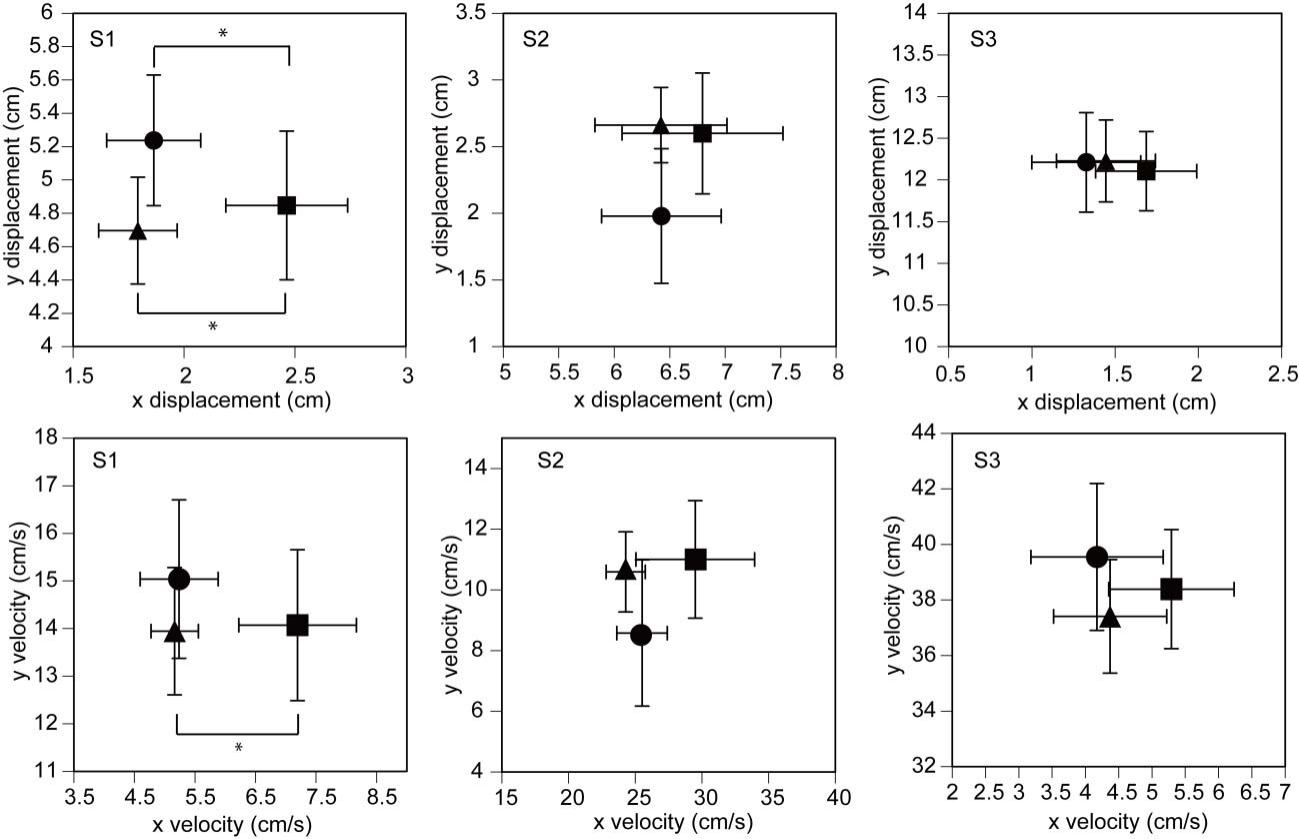
The amplitude (upper figures) and velocity (lower figures) of COP displacement for the asymmetry analysis. The data points indicate the means, and the error bars indicate the standard error of the mean. The triangle points indicate the means in the L condition, the circle points indicate the means in the NI condition, and the square points indicate the means in the R condition. Asterisks indicate significant differences (p < 0.05)

The x displacement in the L condition tended to be larger than that in the NI and R conditions (Figure 3). In particular, the x displacement in the S1 period was significantly different among the three conditions [F(2,20) = 6.619, p = 0.006]. A post-hoc test revealed that the x displacement in the S1 period in the L condition was significantly larger than that in the R and NI conditions (p<0.05). In contrast, the x displacement was not significantly different among the three conditions in the S2 [F(2,20) = 0.593, p = 0.562] or S3 period [F(2,20) = 0.766, p = 0.478]. The y displacement was not significantly different among the three conditions in the S1 [F(2,20) = 1.403, p = 0.269], S2 [F(2,20) = 2.072, p = 0.152] or S3 period [F(2,20) = 0.061, p = 0.941].

The x velocity in the L condition tended to be larger than that in the NI and R conditions. In particular, the x velocity in the S1 period was significantly different among the three conditions [F(2,20) = 4.410, p = 0.026]. A post-hoc test revealed that the x displacement in the S1 period in the L condition was significantly larger than that in the R condition (p<0.05). In contrast, the x velocity was not significantly different among the three conditions in the S2 [F(2,20) = 1.730, p = 0.203] or S3 period [F(2,20) = 0.894, p = 0.425]. The y velocity was not significantly different among the three conditions in the S1 [F(2,20) = 0.404, p = 0.673], S2 [F(2,20) = 1.172, p = 0.330] or S3 period [F(2,20) = 0.871, p = 0.434].

### Leg preference analysis of the COP

**Figure 4:**
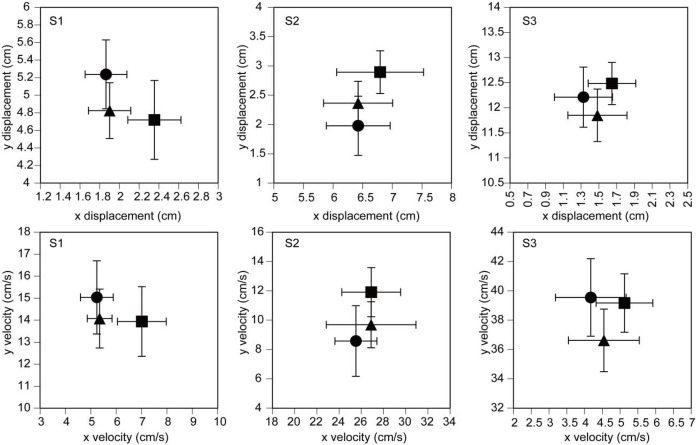
The amplitude (upper figures) and velocity (lower figures) of COP displacement for the leg preference analysis. The data points indicate the means, and the error bars indicate the standard errors of the mean. The triangle points indicate the means in the P condition, the circle points indicate the means in the NI condition, and the square points indicate the means in the NP condition

The x displacement in the NP condition tended to be larger than that in the NI and P conditions (Figure 4). However, the x displacement was not significantly different among the three conditions in the S1 [F(2,20) = 2.810, p = 0.084], S2 [F(2,20) = 0.587, p = 0.565], or S3 period [F(2,20) = 0.575, p = 0.572]. The y displacement was also not significantly different among the three conditions in the S1 [F(2,20) = 1.345, p = 0.283], S2 [F(2,20) = 3.419, p = 0.053], or S3 period [F(2,20) = 1.647, p = 0.218].

The x velocity was not significantly different among the three conditions in the S1 [F(2,20) = 2.951, p = 0.075], S2 [F(2,20) = 0.127, p = 0.882] or S3 period [F(2,20) = 0.554, p = 0.583].

The y velocity was also not significantly different among the three conditions in the S1 [F(2,20) = 0.405, p = 0.673], S2 [F(2,20) = 2.182, p = 0.139], or S3 period [F(2,20) = 2.138, p = 0.144].

## Discussion

### Leg preference analysis

Neither the onset latency of APA, the duration of each period of APA, nor the amplitude and velocity of COP displacement was significantly different among the P, NP, and NI conditions. Thus, our results failed to support our hypothesis that the process of choosing the initial swing leg affected APA during gait initiation. Lateral displacement of the center of gravity during APA was significantly different between the NP and P conditions (Yiou and Do, 2010), and ground reaction force during APA was significantly different between the NP and P conditions (Dessery et al., 2011). However, our present findings were consistent with the previous finding that lateral COP displacement was not significantly different between the NP and P conditions (Yiou and Do, 2010). According to our present findings, it remains inconclusive whether lateral COP displacement during gait initiation depends on choosing the process of the initial swing leg.

### Asymmetry analysis

The main finding of the present study was that the x displacement in the S1 period in the L condition was larger than that in the R and NI conditions. The duration of the S1 period was not significantly different among the three conditions, but the x velocity in the S1 period in the L condition was higher than that in the R condition. Thus, the higher velocity of the COP displacement must be the cause of the greater COP displacement in the S1 period in the L condition. Such asymmetry during the gait has been found; the amplitude of vertical ground reaction force in the left stance phase was larger than that in the right stance phase (Crowe et al., 1993), indicating that the gait and gait initiation may share a common asymmetrical feature of motor control. The COP displacement in the S1 period has been shown to decrease in patients with Parkinson’s disease (Burleigh-Jacobs et al., 1997; Halliday et al., 1998; Dibble et al., 2004; Okada et al., 2011-a). Accordingly, the S1 period must be partially under the control of the supraspinal neural system. Thus, asymmetrical displacement of the COP in the S1 period originates at least partially from the asymmetrical strategy of supraspinal control of gait initiation.

Asymmetrical APA during gait initiation may reflect physiological asymmetry of the legs; e.g., asymmetry of plantar flexor muscle strength between the ankles (Damholt and Termansen, 1978). It has been speculated that the left leg plays a role in supporting weight and the right leg plays a role in propulsion during the gait (Hirasawa, 1981; Sadeghi et al., 2001). The displacement of the COP to the initial swing side in the S1 period when initiating the gait reflects the weight shift to the initial swing leg. Thus, the relatively large displacement of the COP to the initial swing side in the S1 period when initiating the gait with the left leg may reflect the relatively predominant supporting function of that leg. The S1 period of APA is reduced in PD patients, who frequently suffer start hesitation of the gait (Okada et al., 2011-a) and therefore, instructing these patients to initiate the gait with the left leg may be a good strategy for improving APA during gait initiation.

### Limitations

There are two limitations in the present study. One is that there was insufficient evidence to support the hypothesis regarding the mechanism underlying asymmetrical COP displacement in the S1 period. We speculated that the physiological asymmetry of the legs is a possible explanation for the asymmetrical COP displacement in the S1 period. However, we did not measure the physiological difference between the legs, which might provide evidence to support the hypothesis. Further studies regarding this issue are needed. The other limitation is that there was insufficient statistical power to reveal the difference in the COP displacement in the S1 period when the gait was initiated with the right leg versus the left leg. The p-value for the ANOVA, examining the differences between the means of the x displacement in the S1 period among the L, R, and NI conditions was very close to the significance level (p = 0.084). Accordingly, our insignificant finding regarding COP displacement in the S1 period in the leg preference analysis may be due to the insufficient sample size of the experiment.

## Conclusions

The lateral displacement of the COP to the initial swing leg before the gait is initiated with the left leg in response to an external cue is larger than that when the gait is initiated with the right leg in response to an external cue, and is also larger than that when the gait is initiated with the leg spontaneously chosen. The weight shift to the initial swing side during gait initiation is asymmetrical when the leg is chosen in response to an external cue.
